# Signal Detection for GSTFIM Systems with Carrier Frequency Offset †

**DOI:** 10.3390/s22072548

**Published:** 2022-03-26

**Authors:** Tingyong Wu, Shiwen Fan, Chengliang Di, Jinwei Ji

**Affiliations:** 1 The Key Laboratory of Science and Technology on Communications, University of Electronic Science and Technology of China, Chengdu 611731, China; 202022220717@std.uestc.edu.cn; 2 The 54th Research Institute of CETC, Shijiazhuang 050081, China; dichengliang@163.com (C.D.); jjw86215@163.com (J.J.)

**Keywords:** minimum mean-square-error (MMSE), message passing (MP), carrier frequency offset (CFO), generalized space-time-frequency index modulation (GSTFIM)

## Abstract

Generalized space-time-frequency index modulation (GSTFIM) inherits the drawbacks of the conventional orthogonal frequency-division multiplex (OFDM), such as being sensitive to carrier frequency offset (CFO). For a robust design against this problem, in this contribution, a novel construction of a message passing (MP)-aided detector is developed for GSTFIM systems to combat the influence of CFO, while offering a flexible tradeoff between transmission performance and computational complexity. Through complexity analysis and simulation results, we demonstrate that, in the context of CFO, with a careful design, the developed MP detector is capable of approaching traditional GSTFIM with maximum likelihood (ML) detection, and of offering better performance at lower complexity compared to its minimum mean-square-error (MMSE)-aided counterpart.

## 1. Introduction

The combination of multiple-input multiple-output (MIMO) and orthogonal frequency division multiplex (OFDM) remains a technology evolution tendency for future wireless communications, toward enhanced data rates, better error performance, time latency, information security, etc. Recently, following the original idea of spatial modulation [1,2,3,4] and index modulation [5,6,7], the structure of generalized space-time-frequency index modulation (GSTFIM) [8] was developed in order to offer a different sparse signal in the space-time-frequency domain. Specifically, the transmission resource in the space-time-frequency domain is divided into 3-dimensional (3D) points, and only some of these points are activated to transmit efficient data symbols, such as the generalized space-time shift keying (GSTSK) symbol in [9], while the other points remain silent. Therefore, the index of the activated 3D points can be utilized to deliver additional information bits. In general, the above-mentioned structure brings two unique advantages as follows. On the one hand, the partially activated construction enhances the robustness to various types of interference, such as inter-antenna interference (IAI), inter-carrier interference (ICI), and inter-symbol interference (ISI). On the other hand, when combining long enough interleavers [10], the space-time-frequency domain diversity can be fully explored toward enhanced bit-error rate (BER) performance. Due to the above advantages, this structure has the potential to adapt future wireless communications [11] and high-speed vehicular and railway communications [12].

Meanwhile, as an evolution of traditional MIMO-OFDM technology, GSTFIM inherits the disadvantages of original OFDM, such as the sensitivity to ICI caused by carrier frequency offset (CFO). Although this problem may be slightly alleviated due to its sparse 3D structure, as described in current research, the influence on GSTFIM caused by CFO remains a huge challenge, especially in the context of high-mobility scenarios. As a result, the performance of original GSTFIM will be degraded seriously when CFO is considered in practical transmission. In the current literature, a series of techniques has been developed as in [13,14,15,16] for traditional MIMO-OFDM systems. Specifically, for spatial modulation OFDM systems, some CFO compensation techniques at the receiver side were also presented such as in [17,18,19]. However, the above structure cannot be directly utilized in the special structure of GSTFIM. For GSTFIM and index modulation, in order to achieve the performance bound of the optimal detector while fully exploring the sparse structure [20], the concept of message-passing (MP) has been widely considered for low-complexity signal detection [21,22,23]. Recently, we conceived a class of minimum mean-square-error (MMSE) detectors [24] with the capability to combat CFO. However, the attained BER performance still requires improvement while the computational complexity is high for practical implementation.

Against the above background, this paper considers the design of a robust detector for enhancing the BER performance of GSTFIM systems, in the context of an iterative process. The detailed contribution is summarized as follows. Firstly, the iterative detection concept is introduced for general GSTFIM detectors. Specifically, the a priori probabilities of the GSTSK symbols are computed for general GSTFIM detectors, then, a priori probabilities of the GSTSK symbols of all the subcarriers are replaced with posterior probabilities, and the detectors continue the iterative process. After several numbers of iterations, the GSFTIM symbols are ultimately reconstructed. Secondly, two kinds of methods are developed for detector construction. The first method is based on a single carrier, where the probability of the subcarriers in the same GSFTIM symbol is compared to determine whether the symbol on each subcarrier is a GSTSK symbol or an all-zero matrix. The second method is based on block carriers, where the GSFTIM symbols are reconstructed by comparing the probabilities of GSFTIM symbols, which are obtained by the sum probability of several subcarriers in the GSFTIM symbol. Lastly, the idea of message-pass is utilized in signal detection in GSTFIM systems with an iterative process. In general, we conclude that the proposed two methods, as single carrier and block carrier processing, can adapt the MMSE detector in [24] toward a flexible detector configuration. Most important of all, by combining MP and an iterative process, we demonstrate that the developed MP-aided detector can approach the BER performance of maximum likelihood (ML), and outperform MMSE-based detectors with reduced computational complexity.

The remainder of this paper is organized as follows. In Section 2, the GSFTIM system is modeled in the context of CFO. Then, in Section 3, MMSE detectors with different CFO compensation styles are described in detail, followed by another class of developed MP detectors with outstanding performance in Section 4. Section 5 discloses the complexity analysis through theoretical analysis, while Section 6 demonstrates the effectiveness of the developed detectors through simulation results. Finally, concluding remarks are drawn in Section 7.

*Notation:*$E\left\{\cdot \right\}$ and $D\left\{\cdot \right\}$ represent the expectation and variance operators, respectively. The N×N identity matrix is denoted by $I_{N}$.

## 2. GSTFIM Systems with CFO

**Figure 1:**
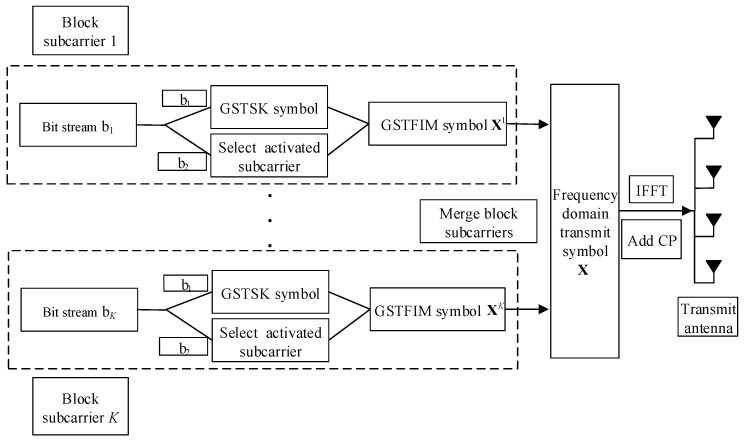
Transmitter structure of the GSTFIM system.

In this paper, we consider a GSTFIM system with $N_{t}$ transmitter antennas, $N_{r}$ receiver antennas, *T* symbol durations, *N* subcarriers, and an *M*-PSK/QAM constellation, as shown in Figure 1. *N* subcarriers are divided into *K* blocks each with $N_{b}$ subcarriers, and only $N_{a}$ out of $N_{b}$ subcarriers are activated. The information bits $b_{k}=b_{1}+b_{2}$, where k={1,2,…,K}, are divided into two parts. The first $b_{1}$ bits are used to generate $N_{a}$ GSTSK symbols as

(1) $$S_{i}^{k}=\sum_{l=1}^{L}s_{l}A_{l}^{k}\in C^{N_{t}\times T},$$

where $i=1,\ldots ,N_{a}$ and *L* is the number of activated dispersion matrices. The second $b_{2}$ bits are employed to select $N_{a}$ activated indices of the antennas out of $N_{b}$, denoted by $I^{k}=\left\{I_{1},I_{2},\ldots ,I_{N_{a}}\right\}$.

Then, we map the GSTSK symbols ${\left[S_{1}^{k},S_{2}^{k},\ldots ,S_{N_{a}}^{k}\right]}^{T}$ to the block matrix as

(2) $$X^{k}={\left[X_{1}^{k},X_{2}^{k},\ldots ,X_{N_{b}}^{k}\right]}^{T}.$$

Specifically, $N_{a}$ elements of $X^{k}$ are expressed as ${\left[S_{1}^{k},S_{2}^{k},\ldots ,S_{N_{a}}^{k}\right]}^{T}$, whose indices are denoted by $I^{k}$, and whose other elements are zeros.

Then, the transmit vector $x=\left[x^{1},x^{2},\ldots ,x^{K}\right]\in C^{NN_{t}\times T}$ in the time domain can be obtained by the IFFT as

(3) $$x=IFFT(\left[X^{1},X^{2},\ldots ,X^{K}\right]).$$

After cyclic prefix (CP) insertion, passing through a wireless channel and CP removal, the time-domain receiver symbols are expressed as $y=\left[y^{1},y^{2},\ldots ,y^{K}\right]\in C^{NN_{r}\times T}$. Then frequency-domain symbols can be obtained by FFT transformation as

(4) $$\left[Y^{1},Y^{2},\ldots ,Y^{K}\right]=FFT\left(\left[y^{1},y^{2},\ldots ,y^{K}\right]\right).$$

We use $e_{m}$ and $f_{n}$ to denote the CFOs between each pair of transmitter and receiver antennas, respectively, where $m=\{1,2,\ldots ,N_{t}\}$ and $n=\{1,2,\ldots ,N_{r}\}$. $e_{m}$ and $f_{n}$ are assumed to be different and remain the same during the symbol duration. Thus, the frequency-domain model with the CFO in the *l*-th subcarrier can be expressed as

(5) $$\begin{array}{c} Y_{l}=\sum_{p=1}^{N}(H_{p}⊙S_{p-l})X_{p}+Z_{l}=\left(H_{l}⊙S_{1}\right)X_{l}+\sum_{p=1,p\neq l}^{N}(H_{p}⊙S_{p-l+1})X_{p}+Z_{l} \end{array},$$

where $l=n_{b}\;+\;N_{b}(k\;\text{-}\;1)\in \left\{1,2,\ldots ,N\right\}$. $X_{l}$ and $X_{p}$ can be expressed as $X_{N_{b}}^{k}$ and $X_{{n^{\prime }}_{b}}^{k^{\prime }}$, respectively, where

(6) $$k^{\prime }=\left\lfloor \frac{p}{N_{b}}\right\rfloor +1,n_{b}^{\prime }=\;\mathrm{mod}\;\left(p,N_{b}\right),$$

$H_{l}\in C^{N_{r}\times N_{t}}$ represents the channel frequency domain impulse response and $Z_{l}\in C^{n_{r}\times T}$ denotes the noise vector, whose elements obey the Gaussian distribution $CN(0,\sigma^{2})$. $S_{p}\in C^{N_{r}\times N_{t}}$ denotes the CFO matrix, which is expressed as

(7) $$S_{p}=\left[\begin{array}{cccc} S_{p}^{\left(1,1\right)} & S_{p}^{\left(1,2\right)} & \ldots & S_{p}^{\left(1,N_{t}\right)}\\ S_{p}^{\left(2,1\right)} & S_{p}^{\left(2,2\right)} & \ddots & S_{p}^{\left(2,N_{t}\right)}\\ \vdots & \ddots & \ddots & \vdots \\ S_{p}^{\left(N_{r},1\right)} & S_{p}^{\left(N_{r},2\right)} & \ldots & S_{p}^{\left(N_{r},N_{t}\right)} \end{array}\right],$$

where p={1,2,…,N} and $S_{p}^{\left(n,m\right)}$ denotes the CFO coefficient [18], which is expressed by

(8) $$S_{p}^{\left(n,m\right)}=\frac{\sin\pi (p+e_{m}+f_{n})}{N\sin\frac{\pi }{N}\left(p+e_{m}+f_{n}\right)}e^{j\pi \left(1-\frac{1}{N}\right)\left(p+e_{m}+f_{n}\right)},$$

where $n\in \{1,2,\ldots ,N_{r}\}$ and $m\in \{1,2,\ldots ,N_{t}\}$.

## 3. Proposed MMSE Detectors for GSTFIM Systems

To mitigate the ICI caused by the CFO and the effect of the channel, Ref. [24] proposed an iterative algorithm to process the receiver symbol $Y_{l}$ based on the MMSE criterion as in [19,25]. In this section, we generalize the MMSE-based detector in [24] in two different ways based upon either a single carrier or block carriers, which are termed “SC-MMSE” and “BC-MMSE”, respectively. First, the MMSE-based detector in [24] is given below.

**Step 1:** Initialize the a priori probability of the transmitter symbol $X_{l}$ as $P\left(X_{l}\;=\;\beta_{i}\right)=\frac{1}{2^{b^{3}}}$, where $\;\;\;b^{3}=\frac{b_{1}}{N_{a}}$ and $\beta_{i}\in \Psi$, Ψ denotes the set of all GSTSK symbols and the $N_{t}\times T$ dimension all-zero matrix. Then, initialize the number of iterations as Σ=1.

**Step 2:** Compute the mean and variance of $X_{l}$ based on the initial a priori probability $P(X_{l}\;=\;\beta_{i})$ as follows

(9) $$\begin{array}{c} E(X_{l})\;=\;\sum_{\beta_{i}\in \Psi }\beta_{i}P\left(X_{l}\;=\;\beta_{i}\right),\\ D(X_{l})\;=\;\sum_{\beta_{i}\in \Psi }\beta_{i}{\beta_{i}}^{H}P\left(X_{l}\;=\;\beta_{i}\right)-E(X_{l})E(X_{l}{)}^{H}. \end{array}$$

**Step 3:** Eliminate the ICI caused by the CFO based on the model (6) as follows

(10) $$W_{l}=Y_{l}-\sum_{p=1,p\neq l}^{N}(H_{p}⊙S_{p-l+1})E\left(X_{p}\right),$$

where $Y_{l}$ and $W_{l}$ can be approximated to

(11) $$Y_{l}=\left(H_{l}⊙S_{1}\right)X_{l}+\sum_{p=l-\frac{G-1}{2},p\neq l}^{l+\frac{G-1}{2}}(H_{p}⊙S_{p-l+1})X_{p}+Z_{l},$$

(12) $$W_{l}=Y_{l}-\sum_{p=l-\frac{G-1}{2},p\neq l}^{l+\frac{G-1}{2}}(H_{p}⊙S_{p-l+1})E\left(X_{p}\right)\;,$$

and G(G≤N) is the number of adjacent subcarriers that affects the *l*-th subcarrier, while the ICI of the other carriers is approximated to zero.

**Step 4:** Apply the MMSE criterion to the model (5) in the subcarrier to obtain

(13) $${\bar{X}}_{l}=C_{l}\left[Y_{l}-\sum_{p=l-\frac{G-1}{2},p\neq l}^{l+\frac{G-1}{2}}(H_{p}⊙S_{p-l+1}\right)E\left(X_{p}\right)]=C_{l}W_{l},$$

where

(14) $$\begin{array}{c} C_{l}=\frac{L}{Q}\left(H_{l}⊙S_{1}\right){]}^{H}\{\frac{L}{Q}\left(H_{l}⊙S_{1}\right){\left(H_{l}⊙S_{1}\right)}^{H}+\\ \;\;\;\;\;\;\;\;\sum_{p=l-\frac{G-1}{2},p\neq l}^{l+\frac{G-1}{2}}(H_{p}⊙S_{p-l+1})D(X_{p}){\left(H_{p}⊙S_{p-l+1}\right)}^{H}\;\;\;\;\;\;\;\;+\sigma^{2}I_{N_{r}\times N_{r}}{\}}^{-1}. \end{array}$$

**Step 5:** Calculate the posterior probability of the transmitter symbols $X_{l}$ as

(15) $$P\left({\bar{X}}_{l}|X_{l}\;=\;\beta_{i}\right)=\psi_{l}e^{-\frac{{∥{\bar{X}}_{l}-\Delta_{l}∥}^{2}}{\omega_{l}^{2}}},$$

where

(16) $$\Delta_{l}=E({\bar{X}}_{l}|X_{l}=\beta_{i})=C_{l}\left(H_{l}⊙S_{1}\right)\beta_{i},$$

(17) $$D_{l}=D({\bar{X}}_{l}|X_{l}=\beta_{i})=\frac{L}{Q}C_{l}\left(H_{l}⊙S_{1}\right)-\frac{L}{Q}C_{l}\left(H_{l}⊙S_{1}\right){\left(H_{l}⊙S_{1}\right)}^{H}C_{l}^{H},$$

(18) $$\omega_{l}^{2}=\frac{trace[D_{l}]}{N_{t}T}.$$

$\psi_{l}$ denotes the normalization coefficient.

**Step 6:** Let Σ=Σ+1 and $P\left(X_{l}\;=\;\beta_{i}\right)=P\left({\bar{X}}_{l}|X_{l}\;=\;\beta_{i}\right)$, and then go back to Step 2. If $\Sigma >\Sigma_{\max}$, the detector proceeds to the next step.

**Step 7:** Reconstruct ${\hat{X}}^{k}$ by the probability $P(X_{l}\;=\;{\mathrm{fi}}_{i})$.

In general, the proposed MMSE detector is only suitable for the situation of Gaussian noise. Under non-Gaussian noise, the calculation of the mean and variance of $X_{l}$ will be inaccurate, resulting in the inaccurate calculation of the subsequent posterior probability. In that case, we could substitute the MMSE criterion in Step 4 by the maximum correntropy criterion [26] to mitigate the inaccuracies. The correntropy between $X_{l}$, ${\bar{X}}_{l}$ with joint distribution $F_{XY}\left(X_{l},{\bar{X}}_{l}\right)$ is defined as

(19) $$V\left(X_{l},{\bar{X}}_{l}\right)=E\left[\kappa \left(X_{l},{\bar{X}}_{l}\right)\right]=\int \kappa \left(X_{l},{\bar{X}}_{l}\right)dF_{XY}\left(X_{l},{\bar{X}}_{l}\right),$$

where E is the expectation operator and $\kappa \left(\cdot ,\;\cdot \right)$ denotes a shift-invariant Mercer kernel, and

(20) $$\kappa \left(X_{l},{\bar{X}}_{l}\right)=G_{\sigma }\left(e\right)=\exp\left(\frac{-e^{2}}{2\sigma^{2}}\right),$$

where $e=\left(X_{l}-{\bar{X}}_{l}\right)$ and σ represents the kernel bandwidth. Then, the correntropy can be estimated by

(21) $$\bar{V}\left(X_{l},{\bar{X}}_{l}\right)=\frac{1}{N}\sum_{i=1}^{N}G_{\sigma }\left(e\left(i\right)\right),$$

where $e\left(i\right)=X_{i}-{\bar{X}}_{i}$.

### 3.1. SC-MMSE Detector

The detail of the SC-MMSE detector is described as follows. For the *l*-th subcarrier, we find the index of the maximum value of the GSTSK symbol in the probability vector $P(X_{l}\;=\;{\mathrm{fi}}_{i})$, which is denoted by $p_{l}$. Repeating the same process for all the *N* subcarriers, the indices can be expressed as

(22) $$P_{SC}=\left\{p_{1},p_{2},\ldots ,p_{N}\right\}.$$

Then, $P_{SC}$ is mapped to *K* blocks to reconstruct the GSTFIM symbol. For the *k*-th block subcarriers, the corresponding carrier indices are $\left\{1+N_{b}\left(k-1\right),2+N_{b}\left(k-1\right),\ldots ,N_{b}k\right\}$, where k=(1,2,…,K). Thus, for the *k*-th block subcarriers, the indices $P_{SC}^{k}$ of the maximum value of the GSTSK symbol are expressed as

(23) $$P_{SC}^{k}=\left\{p_{\left\{1+N_{b}\left(k-1\right)\right\}},p_{\left\{2+N_{b}\left(k-1\right)\right\}},\ldots ,p_{N_{b}k}\right\}.$$

Let $I^{k}$ be the corresponding probability set of $P_{SC}^{k}$ and $\Delta_{k}$ be the indices of the maximum $N_{a}$ values in $I^{k}$. That is, $\Delta_{k}$ records the indices of the activated subcarriers in the *k*-th block.

In general, the estimated GSTFIM symbol ${\hat{X}}^{k}={\left[{\hat{X}}_{1}^{k},{\hat{X}}_{2}^{k},\ldots ,{\hat{X}}_{N_{b}}^{k}\right]}^{T}$ in the *k*-th block can be reconstructed as follows. For ${\hat{X}}_{n_{b}}^{k}\left(n_{b}=1,\ldots ,N_{b}\right)$, if $n_{b}$ belongs to set $\Delta_{k}$, then ${\hat{X}}_{n_{b}}^{k}$ is equal to the GSTSK symbol. Moreover, this GSTSK symbol is obtained by the probability in $I^{k}$ corresponding to $\Delta_{k}$. However, if $n_{b}$ does not belong to set $\Delta_{k}$, ${\hat{X}}_{n_{b}}^{k}$ is estimated to be an all-zero matrix. Traversing $n_{b}$ from 1 to $N_{b}$, we can obtain the estimated GSTFIM symbol ${\hat{X}}^{k}$.

### 3.2. BC-MMSE Detector

The BC-MMSE detector is detailed as follows. For the *k*-th block, the GSTFIM symbol $X^{k}$ has $2^{b_{k}}$ possible values $X=\{E_{1},E_{2},\ldots ,E_{2^{b_{k}}}\}$. For a specific symbol $E_{n}\left(n=1,\ldots ,2^{b_{k}}\right)$, the index set of the GSTSK symbol in $E_{n}$ is denoted by $A=\{a_{1},a_{2},\ldots ,a_{N_{a}}\}$, and the index set of the all-zero matrix is $C=\{c_{1},c_{2},\ldots ,c_{\left(N_{b}-N_{a}\right)}\}$. As mentioned in Section 2, the mapping between the $n_{b}$-th subcarrier of the *k*-th block and the *l*-th of all the *N* subcarriers is $l=n_{b}\;+\;N_{b}(k\;-\;1)$. Thus, the index set A of the GSTSK symbol in all the *N* subcarriers is $A\;+\;N_{b}(k\;-\;1)\;=\;\left\{g_{1},\;\;\;\;g_{2},\;\;\;\ldots ,\;\;\;g_{N_{a}}\right\}$, and the index set E of the zero matrix in all the *N* subcarriers is $C\;+\;N_{b}(k\;-\;1)\;=\;\left\{h_{1},\;\;\;h_{2},\;\;\;\ldots ,\;\;\;\;h_{\left(N_{b}-N_{a}\right)}\right\}$.

According to the probability vector $P(X_{l}\;=\;{\mathrm{fi}}_{i})$, for the GSTSK symbols whose indices are $\left\{g_{1},\;\;\;\;g_{2},\;\;\;\;\;\ldots ,\;\;\;\;g_{N_{a}}\right\}$, their probability vector is expressed as

(24) $$P_{SC-1}=\left\{p_{i_{1}}^{g_{1}},p_{i_{2}}^{g_{2}},\ldots ,p_{i_{N_{a}}}^{g_{N_{a}}}\right\},$$

where $i_{1}\in \left\{1,2,\ldots ,2^{b_{3}}\right\}$ indicates the index of the GSTSK symbol corresponding to index $g_{1}$ in all the $2^{b_{3}}$ possible GSTSK symbols, while $p_{i_{1}}^{g_{1}}$ denotes the $i_{1}$-th value of the probability vector $P(X_{g_{1}}\;=\;{\mathrm{fi}}_{i})$. However, for the all-zero matrices whose indices are $\left\{h_{1},\;\;\;\;\;h_{2},\;\;\;\;\;\ldots ,\;\;\;\;h_{\left(N_{b}-N_{a}\right)}\right\}$, their probability vector is expressed as

(25) $$P_{SC-2}=\left\{p_{\left(2^{b_{3}}+1\right)}^{h_{1}},p_{\left(2^{b_{3}}+1\right)}^{h_{2}},\ldots ,p_{\left(2^{b_{3}}+1\right)}^{h_{\left(N_{b}-N_{a}\right)}}\right\}$$

where $p_{\left(2^{b_{3}}+1\right)}^{h_{1}}$ is the $\left(2^{b_{3}}+1\right)$-th element of the probability vector $P(X_{h_{1}}\;=\;{\mathrm{fi}}_{i})$. Thus, the probability corresponding to the *n*-th symbol in the *k*-th block is

(26) $$\Theta_{n}^{k}=sum\left(P_{SC-1}+P_{SC-2}\right).$$

The probability $\Theta^{k}$ of $X^{k}$ is obtained by traversing *n* from 1 to $2^{b_{k}}$:

(27) $$\Theta^{k}=\left\{\Theta_{1}^{k},\Theta_{2}^{k},\ldots ,\Theta_{2^{b_{k}}}^{k}\right\}.$$

We denote the index of the max probability in $\Theta^{k}$ as *o*, thus $X^{k}$ is estimated as ${\hat{X}}^{k}=E_{o}$.

In general, we propose two detectors SC-MMSE and BC-MMSE to reconstruct the GSTFIM symbol. The difference is that the SC-MMSE detector is realized by comparing the probability of the single carrier, while its BC-MMSE counterpart compares the probabilities of block carriers. The SC-MMSE detector has a lower complexity, but its performance is inferior due to the deletion of information. The BC-MMSE detector takes into account the structure of the GSTFIM symbol, and thus it offers better performance.

It is worth noting that the proposed SC-MMSE and BC-MMSE detectors are different from those in [19] in the following two aspects. (a) The detector in [18] is conceived for SM-OFDM systems, which are derived on the vector-by-vector-based operation. By contrast, the proposed detectors target GSTFIM systems, and are derived from spatial- and time-domain-based matrix operations. Specifically, the mean and variance of all GSTFIM transmitter symbols are calculated based on matrix $X_{l}\in C^{N_{t}\times T}$ in (10) rather than on vector $x_{k}\in C^{N_{t}\times 1}$. In addition, the set of $X_{l}$ includes an all-zero matrix, which does not exist in SM-OFDM systems. (b) In the detector of [18], the posterior probability $P({\hat{x}}_{k}|x_{k}=\alpha_{i})$ of the estimated symbol ${\hat{x}}_{k}$ on the *k*-th subcarrier obtained by the MMSE criterion is utilized to reconstruct the detected SM symbol by comparing the posterior probability. By contrast, in the proposed SC-MMSE detector, the posterior probability $P({\bar{X}}_{l}|X_{l}\;=\;\beta_{i})$ is first employed to decide the possible GSTSK symbol for all the subcarriers, and then the activated subcarrier is obtained by comparing posterior probabilities of the possible GSTSK symbols. However, in the proposed BC-MMSE detector, the posterior probability of block carriers is divided into two parts, namely the posterior probability of the GSTSK (activated) symbol and the posterior probability of a zero matrix, which are obtained from the posterior probability $P({\bar{X}}_{l}|X_{l}\;=\;\beta_{i})$. Then, the posterior probability of block carriers is obtained as the sum of the above two posterior probabilities. Meanwhile, compared to our former work in [24], the original MMSE concept is divided to two detailed implementation styles, i.e., SC- and BC-aided MMSE, for flexible configuration. However, in the next section, we will further propose a class of new MP detectors with better performance.

## 4. Proposed Detectors Based on the MP Criterion

**Figure 2:**
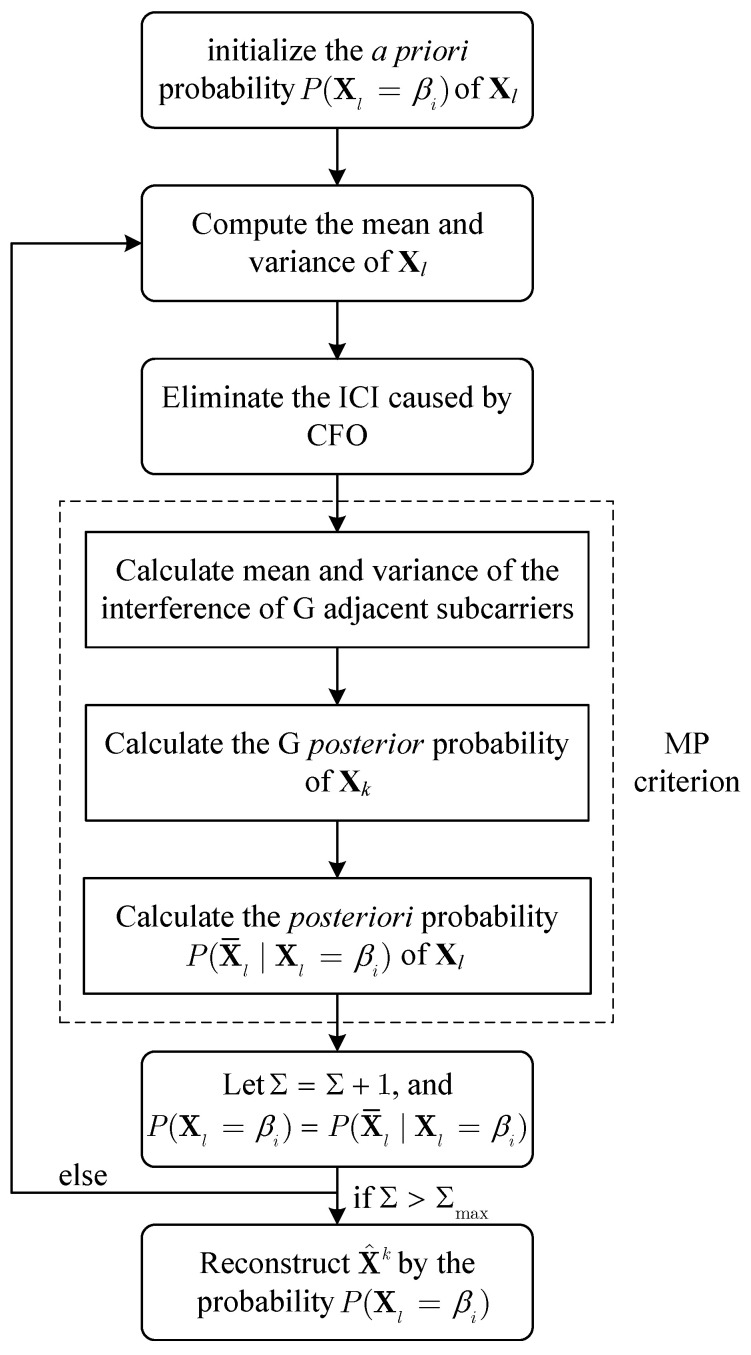
Block diagram of the MP-criterion-based detector.

In this section, the MP-based [27,28,29,30] detectors as shown in Figure 2 are proposed to achieve a better performance. According to the CFO model in (11), the receiver symbol $Y_{l}$ can be regarded as the sum of *G* adjacent symbols $X_{p}$ of the *l*-th subcarrier, where p=(l−(G−1)/2,l+(G−1)/2). In other words, for the *k*-th subcarrier, its transmitter symbol $X_{k}$ has come out *G* times. Thus, these *G* CFO models (11) can be considered as a diversity model of $X_{k}$. Assume that index *h*, which represents one of these *G* adjacent subcarriers around subcarrier *k*, satisfies the condition of $\;\;\;\left|h-k\right|⩽\left(G-1\right)/2$. Thus these diversity models of $X_{k}$ can be described by (28).

(28) $$Y_{h}-\left(H_{k}⊙S_{k-h+1}\right)X_{k}=\sum_{m=h-(G-1)/2,m\neq k}^{h+(G-1)/2}(H_{m}⊙S_{m-h+1})X_{m}+Z_{h}.$$

(29) $$u_{h}^{k}=E\left(\sum_{m=h-(G-1)/2,m\neq k}^{h+(G-1)/2}(H_{m}⊙S_{m-h+1})X_{m}+Z_{h}\right)=\sum_{m=h-(G-1)/2,m\neq k}^{h+(G-1)/2}(H_{m}⊙S_{m-h+1})E\left(X_{m}\right)$$

(30) $$v_{h}^{k}=D\left(\sum_{m=h-(G-1)/2,m\neq k}^{h+(G-1)/2}(H_{m}⊙S_{m-h+1})X_{m}+Z_{h}\right)=\sum_{m=h-(G-1)/2,m\neq k}^{h+(G-1)/2}(H_{m}⊙S_{m-h+1})D\left(X_{m}\right){\left(H_{m}⊙S_{m-h+1}\right)}^{H}+\sigma^{2}I_{N_{r}}$$

Then, the mean $u_{h}^{k}$ and variance $v_{h}^{k}$ of the interference term on the right-hand side of (28) are calculated in (29) and (30), respectively, where the mean $E(X_{m})$ and variance $D(X_{m})$ of $X_{m}$ are calculated using the a priori probability $P(X_{m}\;=\;{\mathrm{fi}}_{i})$ of $X_{m}$ and the calculation is shown in (9).

According the to the MP criterion, the posterior probability of $X_{k}$ can be calculated by the above mean and variance as

(31) $$P_{h}\left(X_{k}\;=\;{\mathrm{fi}}_{i}\right)\propto \exp\left(\frac{\left|\right|Y_{h}-\left(H_{k}⊙S_{k-h+1}\right)X_{k}-u_{h}^{k}{\left|\right|}_{F}^{2})}{trace\left(v_{h}^{k}\right)/N_{r}}\right),$$

where trace(·) denotes the sum of the diagonal elements of a matrix. The overall posterior probability of $X_{k}$ can be obtained by combining the above *G* posterior probabilities in (28) as follows

(32) $$P\left(X_{k}\;=\;{\mathrm{fi}}_{i}\right)\;=\;C_{k}\prod_{\left|h-k\right|⩽\left(G-1\right)/2}P_{h}\left(X_{k}\;=\;{\mathrm{fi}}_{i}\right)$$

where $C_{k}$ is a normalization coefficient. Then the probability $P\left(X_{k}\;=\;{\mathrm{fi}}_{i}\right)$ is used in (10) for the next iteration. In general, the iterative process can be handled as Steps 1–7 using (9) and (25)–(29). After several iterations, similar to Step 7 of the MMSE-based detector in Section 3, the estimated GSTFIM symbol ${\hat{X}}^{k}$ is reconstructed by the probability $P\left(X_{k}\;=\;{\mathrm{fi}}_{i}\right)$. Here, the two MP detectors based on the single carrier and block carriers are dubbed “SC-MP” and “BC-MP”, respectively.

The proposed SC-MP and BC-MP detectors are different from the detector of [30] in the following two aspects. (a) The detector of [30] is conceived for generalized space-and-frequency index modulation (GSFIM) systems, and is derived from spatial- and frequency-domain-based matrix operations. The proposed detectors are conceived for GSTFIM systems, and are derived on spatial- and time-domain-based matrix operation. Specifically, the mean and variance of the GSTFIM transmitter symbols in the *l*-th subcarrier are calculated based on matrix $X_{l}\in C^{N_{t}\times T}$ in (9) rather than matrix $X_{g}\in C^{N_{t}\times N_{f}}$. That is, the basic unit is a spatial- and time-domain symbol in the proposed detectors, while it is a spatial- and frequency-domain symbol in [30]; (b) The GSTFIM transmit symbols can be reconstructed by combining three-dimensional information including $N_{r}$ antennas, $N_{b}$ subcarriers, and *T* symbol durations, rather than only combining $N_{r}$ antennas and $N_{f}$ subcarriers in [30], and the set of $X_{l}$ includes the zero matrix, which is not existent in [30]. Specifically, in the detector of [30], the posterior probability of the GSFIM symbol $X_{g}\in C^{N_{t}\times N_{f}}$ obtained via the MP criterion is directly utilized to reconstruct the detected GSFIM symbol by comparing the posterior probabilities. However, in the proposed SC-MP detector, the posterior probability $P\left(X_{k}\;=\;{\mathrm{fi}}_{i}\right)\;$ is used to first select a possible GSTSK symbol for all the subcarriers, and then the activated subcarrier is obtained by comparing the posterior probability of the possible GSTSK symbol in different subcarriers. In the proposed BC-MP detector, the probability of block carriers is calculated by two parts, namely the probability of the GSTSK (activated) symbol and the all-zero matrix, which are obtained by the posterior probability $P\left(X_{k}\;=\;{\mathrm{fi}}_{i}\right)$. Finally, the sum of the probabilities of the GSTSK symbol and all-zero matrix is considered as the probability of the block subcarriers. However, due to the unique advantage of MP, we will disclose the performance improvement of the MP detector over its counterpart MMSE-based one, but with reduced computational complexity.

## 5. Complexity Analysis

In this section, the complexity of the proposed detectors is analyzed in terms of the number of real-valued flops [12], where a real-valued multiplication and a real-valued addition are both considered as one real-valued flop. The complexities of the proposed SC-MMSE, BC-MMSE, SC-MP, and BC-MP detectors are given in (33), (34), (35), and (36), respectively.

(33) $$\begin{array}{c} C_{SC-MMSE}\;=\;\underset{\left(8\right)}{\underset{︸}{10N_{r}N_{t}T}}+\underset{\left(10\right)}{\underset{︸}{(4N_{t}T2^{b_{3}}N-2N_{t}TN)}}+\underset{\left(10\right)}{\underset{︸}{\left(8T+2\right)N_{t}^{2}2^{b_{3}}+\left(8T-2\right)N_{t}^{2}}}+\underset{\left(11\right)}{\underset{︸}{GNN_{t}N_{r}\left(6+8T\right)}}\\ \;\;\;\;\;\;\;\;\;\;\;\;\;\;\;\;\;\;\;\;\;\;\;\;\;\;\;\;\;\;\;\;\;\;\;\;\;\;\;\;\;\;\;\;+\underset{\left(21\right)}{\underset{︸}{\{G\left(8N_{t}^{2}N_{r}+8N_{r}^{2}N_{t}-2N_{t}N_{r}\right)+4N_{r}^{3}+8N_{r}^{2}\}N}}+\underset{\left(21\right)}{\underset{︸}{\{9N_{r}+8N_{t}+1\}N}}+\underset{\left(20\right)}{\underset{︸}{(8N_{r}-2)N_{t}TN}}\\ \;\;\;\;\;\;\;\;\;\;\;\;\;\;\;\;\;\;\;\;\;\;\;\;\;\;\;\;\;\;\;\;\;\;\;\;\;\;\;\;\;\;\;+\underset{\left(22\right)}{\underset{︸}{(8N_{r}-2)N_{t}^{2}N}}+\underset{\left(23\right)}{\underset{︸}{(8N_{t}+8N_{r}+2)N_{t}^{2}N}}+\underset{\left(24\right)}{\underset{︸}{N_{t}N\;+\;N}}+\underset{\left(25\right)}{\underset{︸}{(8N_{t}^{2}T+4N_{t}T+3)2^{b_{3}}N-N}} \end{array}.$$

(34) $$C_{BC-MMSE}\;=\;C_{SC-MMSE}+\underset{\left(30\right)}{\underset{︸}{2^{b_{k}}K\left(N_{b}-1\right)}}$$

(35) $$\begin{array}{c} C_{SC-MP}\;=\;\underset{\left(8\right)}{\underset{︸}{10N_{r}N_{t}T}}+\underset{\left(33\right)}{\underset{︸}{(4N_{t}T2^{b_{3}}-2N_{t}T)N}}+\underset{\left(33\right)}{\underset{︸}{\{\left(6N_{r}N_{t}+8N_{r}N_{t}T\right)\left(G-1\right)-2N_{r}T\}NG}}\\ \;\;\;\;\;\;\;\;\;\;\;\;\;\;\;\;\;\;\;\;\;\;\;\;\;\;\;\;\;\;\;\;\;\;\;+\underset{\left(34\right)}{\underset{︸}{\{\left(8T+2\right)N_{t}^{2}2^{b_{3}}+\left(8T-2\right)N_{t}^{2}\}N}}+\underset{\left(34\right)}{\underset{︸}{\left\{(8N_{r}N_{t}^{2}-2N_{r}N_{t}+8N_{r}^{2}N_{t}\right)\left(G-1\right)-2N_{r}^{2}+N_{r}\}NG}}\\ \;\;\;\;\;\;\;\;\;\;\;\;\;\;\;\;\;\;\;\;\;\;\;\;\;\;\;\;\;\;\;\;\;\;+\underset{\left(35\right)}{\underset{︸}{\left\{6N_{r}N_{t}+2N_{r}T+(8N_{r}N_{t}T+4N_{r}T+N_{r}\right)2^{b_{k}}\}NG}}+\underset{\left(36\right)}{\underset{︸}{N\{2^{b_{3}}\left(G+1\right)-1\}}} \end{array}$$

(36) $$C_{BC-MP}\;=\;C_{SC-MP}+2^{b_{k}}K\left(N_{b}-1\right)$$

In fact, the complexity of the proposed SC-MMSE detector is due mainly to (7), (9), (10), and (13)–(18). More specifically, the number of flops of $E(X_{l})$ in (9) is given as follows. $\beta_{i}P\left(X_{l}\;=\;\beta_{i}\right)$ needs $2N_{t}T$ flops and this operation is executed $2^{b_{3}}N$ times. $\sum_{\beta_{i}\in \Psi }\beta_{i}P\left(X_{l}\;=\;\beta_{i}\right)$ needs $2N_{t}T\left(2^{b_{3}}-1\right)$ flops and this operation is executed *N* times. Therefore, the number of flops required for (9) is $4N_{t}T2^{b_{3}}N-2N_{t}TN$, while the number of flops for the other equations can be calculated in a similar way.

## 6. Simulation Results

In this section, the BERs of the proposed SC-MMSE, BC-MMSE, SC-MP, and BC-MP detectors are simulated. The maximum likelihood detector without the CFO (“ML-IDEAL”) and the ML detector with the CFO (“ML-WORST”) are given as two baselines. Moreover, QPSK modulation and the Extended Vehicular A (EVA) channel model [31] are employed in the GSTFIM system.

**Figure 3:**
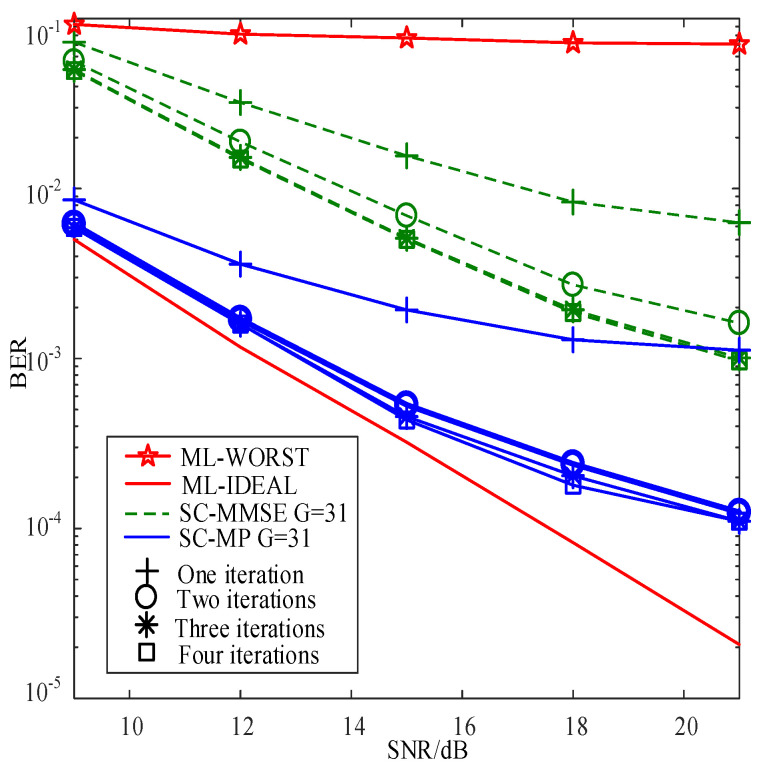
BER comparisons of the proposed SC-MMSE and SC-MP detectors for the GSTFIM systems with $N_{t}=2$ and $N_{r}=2$.

**Figure 4:**
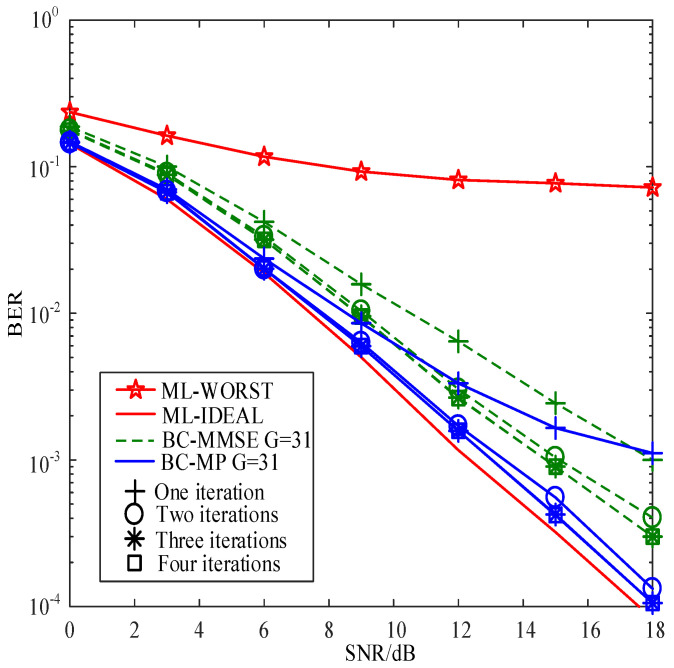
BER comparisons of the proposed BC-MMSE and BC-MP detectors for the GSTFIM systems with $N_{t}=2$ and $N_{r}=2$.

**Figure 5:**
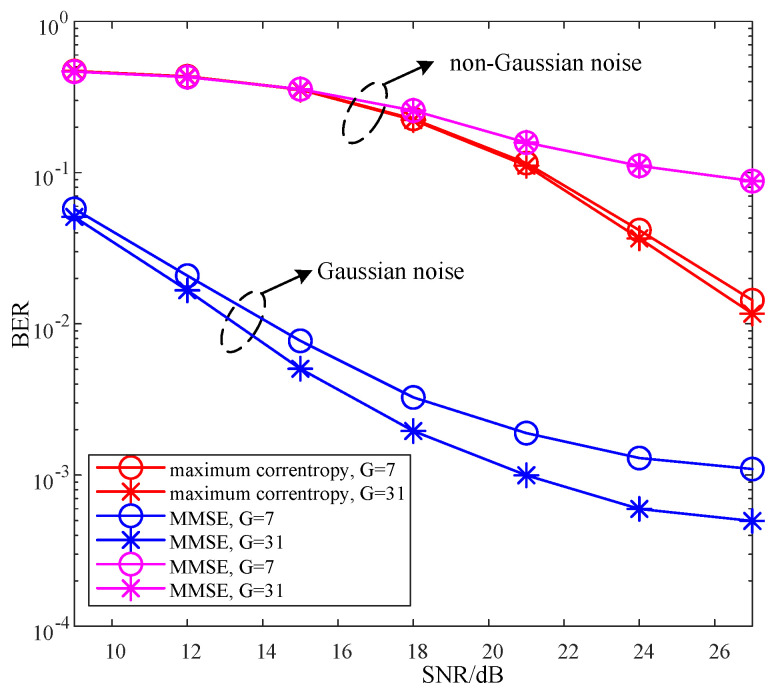
BER comparisons of the proposed SC-MMSE detectors for the GSTFIM systems with $N_{t}=2$ and $N_{r}=2$ under Gaussian noise and non-Gaussian noise.

Figure 3 compares the BERs of the proposed SC-MMSE and SC-MP detectors for the GSTFIM system with different numbers of iterations, while Figure 3 compares the BERs of the proposed BC-MMSE and BC-MP detectors. The specific GSFTIM system parameters in Figure 3 and Figure 4 are as follows: $N_{t}=2,N_{r}=2,N=128,K=64,N_{b}=2,N_{a}=1,L=2,$G=31, and T=2. The CFOs $e_{m}$ and $f_{n}$ are independent and random from frame to frame following a uniform distribution over the range −0.2 to 0.2, and the length of the CP is 32. As can be observed from Figure 3 and Figure 4, the performances of the proposed four detectors are converged in three iterations. Moreover, the convergence behaviors of the proposed SC-MP and BC-MP detectors are similar to that of the ML-IDEAL detector. Meanwhile, Figure 5 compares the BERs of the proposed SC-MMSE detectors for the GSTFIM system with four iterations under different Gaussian noise types, i.e., Gaussian noise and non-Gaussian noise. The specific GSFTIM system parameters in Figure 5 are same as those in Figure 3. As can be seen from Figure 5, under non-Gaussian noise, the proposed MMSE-criterion-based detector exhibits worse performance. In this situation, the maximum-correntropy-based detector can slightly improve the BER performance. However, the performance is still much worse than that under Gaussian noise. This is mainly because there is a deviation in the calculation of the mean and variance of $X_{l}$ under non-Gaussian noise, resulting in the inaccurate calculation of the posterior probability.

**Figure 6:**
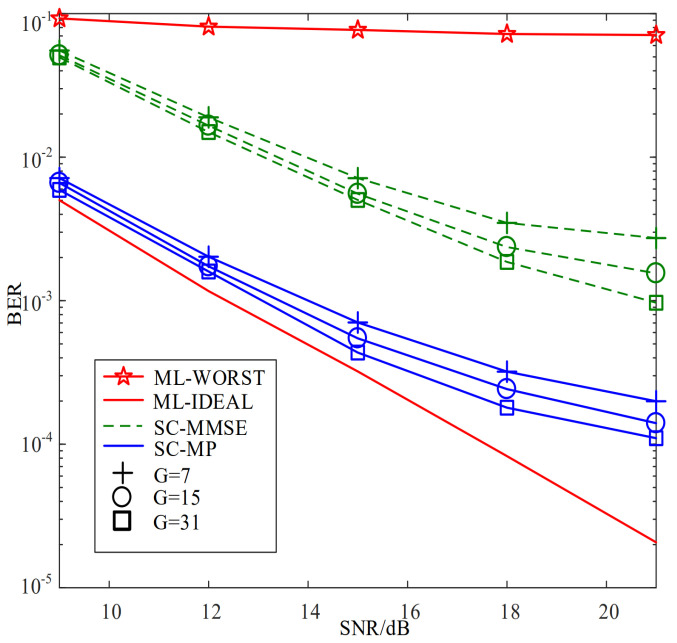
BER comparisons of the proposed SC-MMSE and SC-MP detectors for the GSTFIM systems with different numbers of effective subcarriers *G* in three iterations.

**Figure 7:**
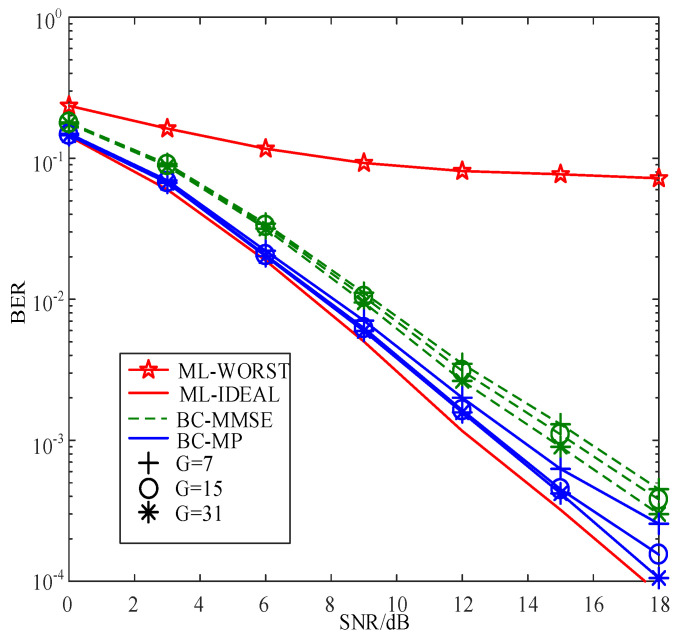
BER comparisons of the proposed BC-MMSE and BC-MP detectors for the GSTFIM systems with different numbers of effective subcarriers *G* in three iterations.

**Figure 8:**
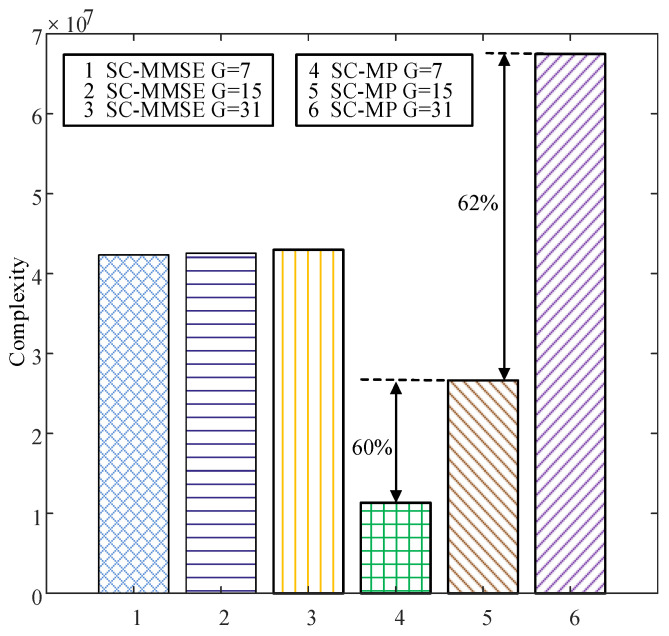
Complexity comparisons of the proposed SC-MMSE and SC-MP detectors for the GSTFIM systems with different numbers of effective subcarriers *G* in three iterations.

**Figure 9:**
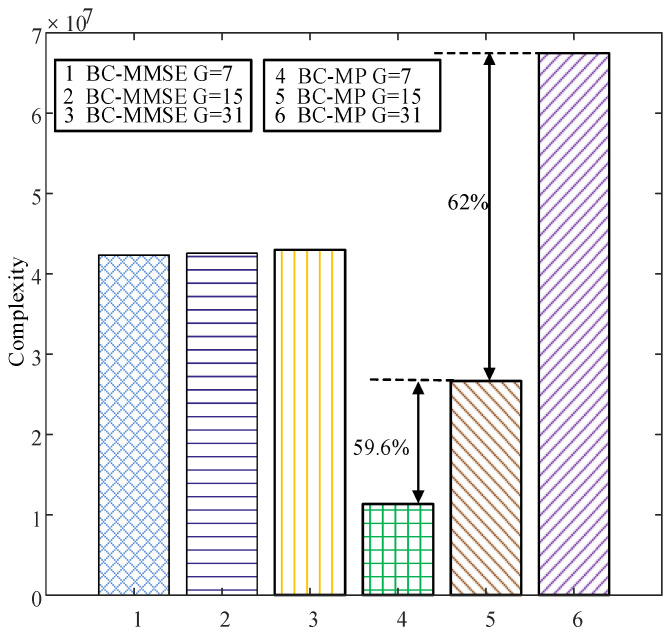
Complexity comparisons of the proposed BC-MMSE and BC-MP detectors for the GSTFIM systems with different numbers of effective subcarriers *G* in three iterations.

**Figure 10:**
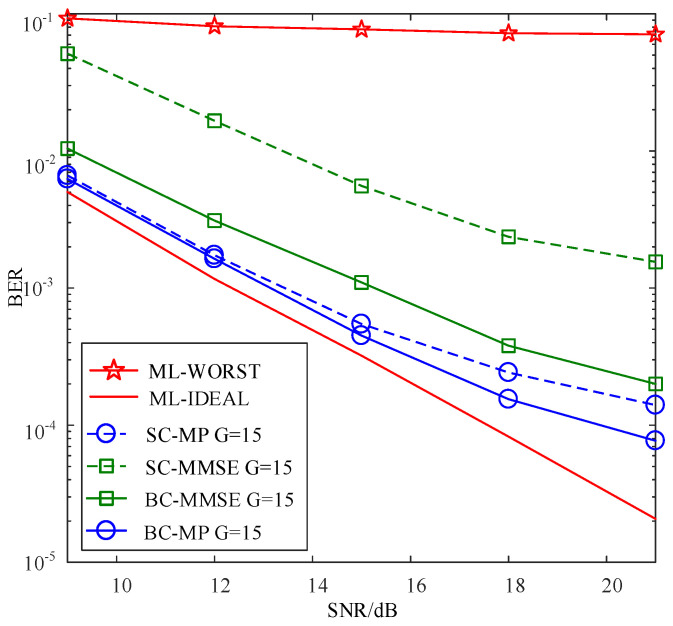
BER comparisons of the proposed SC-MMSE, SC-MP, BC-MMSE, and BC-MP detectors for the GSTFIM systems with $G=15,N_{t}=2$, and $N_{r}=2$ in three iterations.

**Figure 11:**
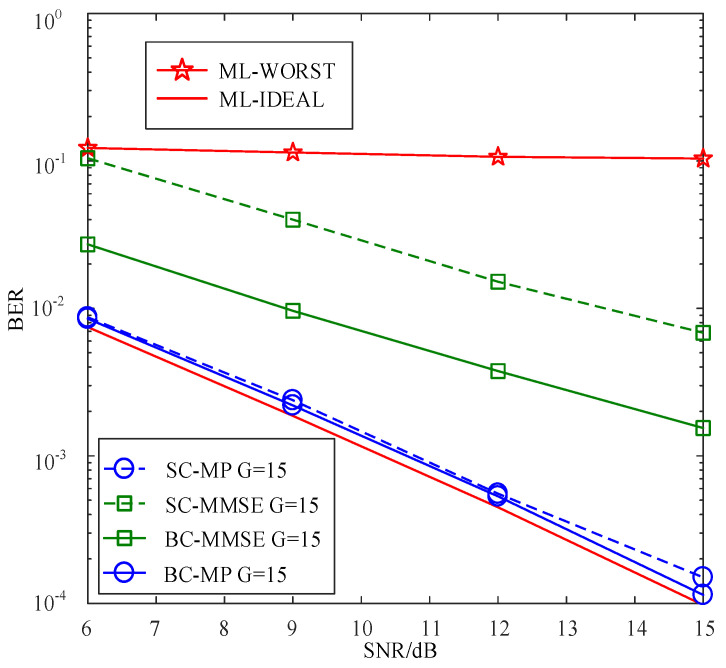
BER comparisons of the proposed SC-MMSE, SC-MP, BC-MMSE, and BC-MP detectors for the GSTFIM systems with $G=15,N_{t}=2$, and $N_{r}=4$ in three iterations.

Figure 6 compares the BERs of the proposed SC-MMSE and SC-MP detectors for the GSTFIM systems with different numbers of effective subcarriers *G* in three iterations, while Figure 7 depicts the BERs of the proposed BC-MMSE and BC-MP detectors. The other system parameters in Figure 6 and Figure 7 are same as those in Figure 3. Figure 8 and Figure 9 show the complexities of Figure 6 and Figure 7, respectively. As can be seen in Figure 6 and Figure 7, the performances of the proposed four detectors become better with the increase in the number of effective subcarriers *G*. However, according to the complexity comparisons in Figure 8 and Figure 9, it can be observed that the complexities of the proposed MMSE-based detectors do not increase much with the increase in the number of effective subcarriers, while that of the proposed MP-based detectors increases significantly when the number of effective subcarriers increases. In order to balance between performance and complexity, we choose the number of effective subcarriers to be G=15 as an example. Then, the BERs of the proposed SC-MMSE, BC-MMSE, SC-MP, and BC-MP detectors with $N_{t}=2$, $N_{r}=2,G=15$ are compared in Figure 10, which shows that the MP-based detectors offer a better performance than their MMSE-based counterparts. Moreover, the detector based on block carriers offers a better performance than the one based on the single carrier due to the fact that the detector based on block carriers is able to maintain the integrity of the system symbols. Finally, the BERs of the proposed four detectors for GSTFIM systems are compared in Figure 11. The specific GSFTIM system parameters of Figure 10 are $N_{t}=2,N_{r}=4,N=64,K=32,N_{b}=2,N_{a}=1,L=1,G=15$, and T=2. The CFOs $e_{m}$ and $f_{n}$ are independent and random from frame to frame following a uniform distribution over the range −0.3 to 0.3, and the length of the CP is 16. The similar trend exhibited in Figure 10 can be found in Figure 11.

## 7. Conclusions

In order to combat ICI caused by CFO in GSTFIM systems, we considered the construction of two kinds of detectors, i.e., MMSE- and MP-based detectors, in an iterative process toward enhanced BER performance. Specifically, based on our former works in [24], the MMSE detector was detailed in two working styles to offer a flexible construction of detection. Most important of all, an MP-based detector was developed for improving the detection permanence compared to its MMSE counterpart. When G is relatively small, the MP-based detector is even able to reduce complexity. Considering that CFO is not the only problem in practical transmission, in future works, we will focus on mitigating the influence of non-ideal time synchronization and non-Gaussian distribution toward a more robust construction of GSTFIM systems.

## Data Availability

Not applicable.
